# Cranial Tibial Wedge Osteotomy in Five Cats with Cranial Cruciate Ligament Rupture

**DOI:** 10.3390/ani16131959

**Published:** 2026-06-25

**Authors:** Fidel San Román-Llorens, Alejandro Blanco, Fidel San Román, Cristina González, Alberto Climent, Julia Laliena, Manuel Alamán, Ana Whyte

**Affiliations:** 1 Department of Animal Pathology, Faculty of Veterinary Science, University of Zaragoza, 50013 Zaragoza, Spain; elbuzondefidel@hotmail.com (F.S.R.-L.);; 2 Hospital Centro Clínico Veterinario de Zaragoza, 50006 Zaragoza, Spain; 3 Hospital de Urgencias Veterinarias de la Región de Murcia, 30110 Murcia, Spain; 4 Department of Animal Medicine and Surgery, Faculty of Veterinary Science, Complutense University of Madrid, 28040 Madrid, Spain

**Keywords:** cruciate ligament rupture, cranial tibial wedge osteotomy, cats, stifle join

## Abstract

Cranial cruciate ligament (CrCL) rupture in cats is less common than in dogs, and its optimal treatment remains a subject of debate. This study aimed to evaluate the application of cranial tibial wedge osteotomy (CTWO) as a dynamic stabilization technique in cats with CrCL rupture, describing the technical aspects and clinical outcomes obtained. Five cases with a confirmed diagnosis of CrCL rupture between 2020 and 2024 were included in this study. All patients were treated with CTWO using specific osteosynthesis locking plates designed for use in dogs and a complementary cerclage wire. Radiographic rechecks were performed at 8 and 12 weeks postoperatively, and clinical evaluations were performed 24 h, 8 weeks, 12 weeks, and 6 months postoperatively in every patient. Successful and complete bone healing of the tibial osteotomy was observed in every case. No intraoperative or postoperative complications related to implants or soft tissues were recorded. All cats achieved complete functional recovery without lameness at the last recheck six months after surgery. The technique was performed without significant technical difficulties, providing adequate stability and favorable clinical outcomes in all cases. These preliminary results support the use of CTWO as an effective surgical alternative for the treatment of CrCL rupture in cats. However, further studies with a larger number of cases and a longer follow-up are required to better evaluate its clinical application, outcomes, and influence on osteoarthritis progression in the long term.

## Simple Summary

Cranial cruciate ligament rupture is an uncommon but clinically relevant condition in cats, associated with pain, joint instability, and the development of osteoarthritis (OA). Several surgical techniques have been described, although there is no clear consensus regarding the optimal treatment in this species. This study evaluates the use of cranial tibial wedge osteotomy (CTWO) as a single surgical technique in five cats diagnosed with cranial cruciate ligament rupture. The biomechanical principle of this technique is based on reducing the tibial plateau angle, thereby neutralizing cranial tibial thrust during weight-bearing activities and achieving dynamic stabilization of the stifle joint without the need for ligament replacement. The procedure was performed using locking plates and a complementary cerclage wire to stabilize the osteotomy. Clinical and radiographic follow-up demonstrated complete bone healing in all cases within 8–12 weeks, without implant-related or soft tissue complications. At six months postoperatively, all cats showed complete functional recovery, with absence of lameness. These preliminary findings suggest that CTWO may represent a safe and effective surgical alternative for the treatment of cranial cruciate ligament rupture in cats. However, further studies with larger sample sizes and longer follow-up periods are needed to confirm these results and evaluate long-term outcomes.

## 1. Introduction

The cranial cruciate ligament (CrCL) prevents cranial displacement and excessive internal rotation of the tibia, in addition to limiting stifle extension [1,2]. Its rupture causes pain, effusion, instability, and, eventually, joint osteoarthritis (OA) [2]. For unknown reasons, the incidence of rupture is much lower in cats than in dogs; it has been postulated that this may be partly because its size in cats is larger compared to the caudal cruciate ligament [2,3]. Regarding its etiology, some authors consider it to have a purely traumatic origin [4], while others distinguish two groups: one of traumatic origin, often associated with rupture of other stifle ligaments, and another without a traumatic history or with minimal trauma [3,5]. The most recent literature indicates that up to 14% of cats may develop bilateral CrCL rupture, supporting the hypothesis of a degenerative etiology [6]. The association between CrCL rupture and meniscal injury has been well described in surgical treatment reports involving arthrotomy [5,7,8].

Therapeutic options are divided into conservative treatments with anti-inflammatory drugs and rest [6,9] and surgical approaches. Extracapsular techniques with prosthetic synthetic reinforcements [3,10,11] or muscle transposition [12], tibial plateau leveling osteotomy (TPLO) [7,13], and tibial tuberosity advancement (TTA) [14,15] have already been reported to treat this pathology in cats. Combined treatment with TPLO and a closed cranial tibial wedge osteotomy (CTWO) was also described in one case presenting a deformity of the proximal tibia with an exaggerated tibial plateau angle of approximately 75 degrees [16]. However, to the best of the authors’ knowledge, only one additional publication describing the application of the CTWO technique as a single surgical technique in feline patients is currently available in the literature. Aleksiewicz et al. described the use of CTWO in ten cats and specifically emphasized the use of a triangular saw guide designed to facilitate the osteotomy procedure, allowing more precise and orthogonal osteotomy cuts and achieving satisfactory clinical outcomes [17].

The main purpose of this study was to describe the surgical technique and its clinical application in cats affected by cranial cruciate ligament rupture. Given the limited number of publications currently available on the use of cTWO in feline patients, the authors sought to contribute additional clinical information and expand the existing knowledge previously reported by Aleksiewicz et al. [17].

## 2. Materials and Methods

### 2.1. Patients

**Figure 1:**
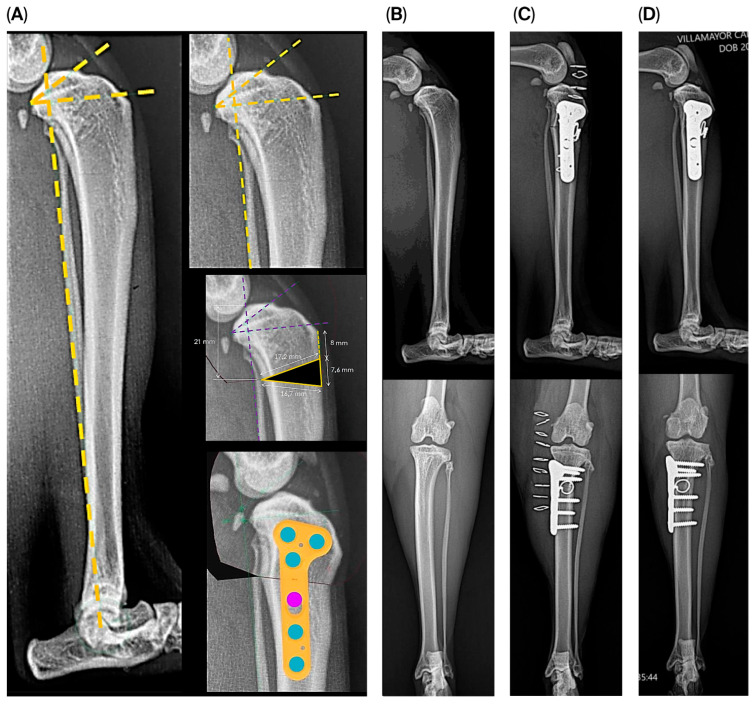
Case 5: Preoperative planning, including TPA measurement, osteotomy planning, and simulation of plate placement once the osteotomy has been reduced (block (**A**)). Preoperative mediolateral and caudocranial projections (**B**). Postoperative views after performing, reducing, and fixing the osteotomy with a CTWO plate and cerclage (**C**), and complete bone healing 12 weeks after surgery (**D**).

Five cats treated between 2020 and 2024 with a diagnosis of cranial cruciate ligament rupture were included. The general clinical examination of all cases was recorded, including anamnesis (duration of lameness, traumatic history, and previous treatments), breed, age, sex, and degree of lameness (grade 0 = no lameness, grade 1 = mild, grade 2 = moderate, grade 3 = severe, grade 4 = non-weight-bearing) [18]. Subsequently, blood analyses (hematology and biochemistry) were performed. Orthopedic and radiographic examinations were carried out by the same observer (FS) under sedation using dexmedetomidine (Dexdomitor^®^, Zoetis, Louvain-la-Neuve, Belgium; 0.0025–0.01 mg/kg IM), butorphanol (Torbugesic^®^, Zoetis, Louvain-la-Neuve, Belgium; 0.2–0.4 mg/kg IM), and ketamine (Ketamidor^®^, Richter Pharma AG, Wels, Austria; 2–5 mg/kg IM). During orthopedic examination, joint swelling, crepitus, and stifle range of motion (ROM) were assessed, and tibial compression and cranial drawer tests were performed. The radiographic study included two orthogonal medio-lateral and caudocranial views of the affected stifle including the tarsal joint (Figure 1B).

OA was subjectively assessed by the authors according to Freire et al. [19]. The patients were defined and classified into radiographic grades by osteoarthritis score (OAS) from 0 to 10 (0 = no identifiable radiographic changes; 1–3 = mild OA; 4–6 = moderate OA; 7–9 = severe OA; 10 = most severe AO).

### 2.2. Preoperative Planning

Radiographs of the affected limb were examined to evaluate limb alignment and determine the preoperative tibial plateau angle (preoperative TPA; Figure 1A) according to the previously described method [16,20,21] using a digital radiography system (Rayence^®^ model 1717SCV, Rayence, Closter, NJ, USA) and commercial veterinary software (Beta Implants App^®,^ version 1.1). CTWO planning followed the method previously reported by Oxley et al. [21]. An isosceles triangle-shaped wedge was positioned as proximally as possible while preserving sufficient bone stock for plate fixation and maintaining an adequate distance distal to the tibial tuberosity of a minimum of 5 mm (Figure 1A). The planned wedge angle varied according to the tibial plateau angle (TPA) to compensate for the greater displacement of the tibial long axis associated with larger wedges, following Oxley et al.’s planned wedge angles based on a preoperative TPA table [21].

### 2.3. Surgical Technique

Patients were premedicated with dexmedetomidine (Dexdomitor^®^, Zoetis; 0.003 mg/kg IV) and methadone (Semfortan^®^, Dechra, Turin, Italy; 0.25 mg/kg IV). General anesthesia was induced with propofol (Propofol-Lipuro^®^, B. Braun Melsungen AG, Melsungen, Germany; 2–4 mg/kg IV) and maintained with inhaled sevoflurane (Sevoflo^®^, Zoetis) in oxygen. Additional perioperative analgesia consisted of meloxicam (Metacam^®^, Boehringer Ingelheim, Berkshire, UK; 0.3 mg/kg IV), and perioperative antibiotic therapy was performed with cefazolin (Cefazolina Normon^®^, Laboratorios Normon, S.A., Madrid, Spain; 22 mg/kg IV); both drugs were administered 45 min before surgery.

All surgical procedures were performed by the same surgeon (FS). A standard medial surgical approach was made to the stifle and proximal third of the tibia. A medial parapatellar mini-arthrotomy was then performed in order to inspect the cruciate ligaments and the medial and lateral menisci to evaluate their integrity. Joint inspection with a meniscal probe, debridement of the torn cranial cruciate ligament fibers, and partial medial meniscectomy when necessary were carried out using a number 11 scalpel blade (Braun^®^, Melsungen, Germany). Intact menisci were left intact. The joint capsule was closed routinely with interrupted simple sutures using polydioxanone 2-0 (PDS II^®^, J&J MedTech, Norderstedt, Germany). Subsequently, the preoperatively planned osteotomy was marked on the medial surface of the proximal tibia using a monopolar scalpel. Subperiosteal elevation was performed caudally and laterally at the osteotomy level, and moistened gauze pads were placed before performing the osteotomy in all cases in order to protect the soft tissue and vascular structures during the osteotomy.

**Figure 2:**
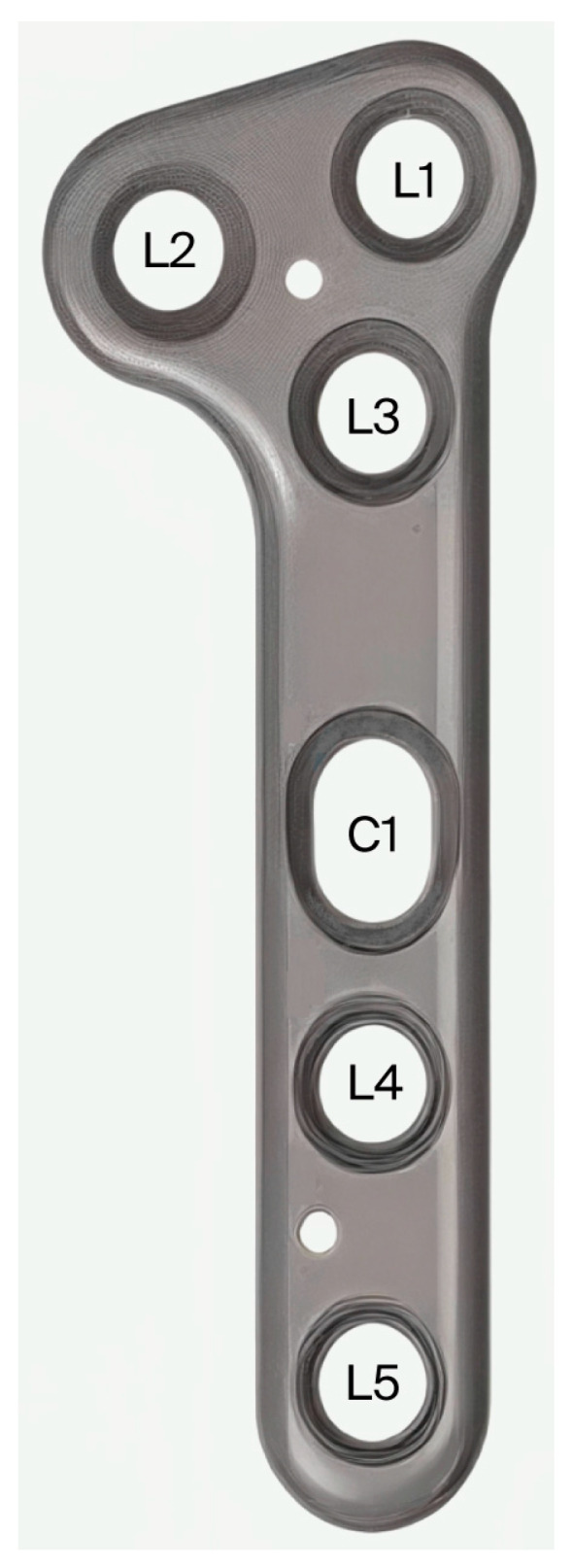
Schema of screw labeling of all 2.0 mm and 2.4 mm CTWO locking plates used in this study. Locking screws are labeled L1–L5 from proximal to distal. The only cortical screw of the plate that allows a dynamic compression function is labelled as C1.

A 0.8 mm orthopedic wire (Veterinary Instrumentation^®^, Sheffield, UK) was placed through 1.1 mm holes positioned cranially, approximately 2–3 mm proximal and distal to the osteotomy surfaces, to reduce and stabilize the wedge before plate application. This cerclage wire was applied in a closed loop fashion with a single medial twist knot and was not removed after osteosynthesis plate placement. Care was taken to avoid positioning the cranio-proximal hole too close to the patellar tendon insertion. Finally, a 2.0 mm or 2.4 mm CTWO locking plate (Beta Implants^®^, Pontevedra, Spain; Figure 2), appropriate to the patient’s size, was placed (Figure 1C). Layered closure was performed routinely after surgical lavage, and a sterile adhesive dressing was maintained for 24 h.

### 2.4. Postoperative Care

Patients were hospitalized for 24 h after surgery with maintenance intravenous fluid therapy, methadone (Semfortan^®^; 0.25 mg/kg IV every 6 h), meloxicam (Metacam^®^; 0.05 mg/kg IV every 24 h), and cefazolin (Cefazolina Normon^®^; 22 mg/kg IV every 12 h). The following day, all patients were discharged with an Elizabethan collar. At home, they received meloxicam (Metacam^®^; 0.05 mg/kg PO every 24 h for 14 days) and cefadroxil (Cefadroxilo Normon^®^, Laboratorios Normon, S.A., Madrid, Spain; 20 mg/kg PO every 24 h for 7 days). Cage rest was prescribed for eight weeks until the first radiographic follow-up.

### 2.5. Clinical Follow-Up

Clinical evaluations were performed immediately before discharge, two weeks after surgery (coinciding with suture removal), and at eight weeks, twelve weeks, and six months postoperatively. During these rechecks, the surgical wound, limb swelling, orthopedic examination, and clinician-assessed lameness grade were evaluated. Owners were also asked to subjectively assess lameness (none = grade 0, mild = grade 1, mild intermittent to moderate = grade 2, severe = grade 3, non-weight-bearing = grade 4) and to rate the patient’s mobility and comfort level during each follow-up after discharge.

### 2.6. Radiographic Follow-Up

The same mediolateral and craniocaudal stifle radiographic views performed preoperatively were repeated postoperatively and at all radiographic follow-ups for every patient (Figure 1B–D). Postoperative radiographs were performed immediately after surgery to evaluate the osteotomy and implant placement, limb alignment, and postoperative tibial plateau angle (postop TPA). Radiographic rechecks were performed eight and twelve weeks postoperatively under sedation with the same anesthetic protocol used for preoperative radiographic examination. Bone healing follow-up was performed by assessing the osteotomy lines cranial and caudal to the CTWO osteosynthesis plate on the mediolateral projection. Bone union was defined as fusion of the osteotomy line, the presence of bridging callus, or disappearance of the osteotomy line. The degree of bone union was scored as follows: 1 = poor union, <25% healing; 2 = acceptable union, 25–50% healing; 3 = good union, >50–75% healing; and 4 = excellent union, >75% healing. OA was subjectively assessed by the authors according to Freire et al. [19]. Based on this study, severity of each radiographic change was graded on a five-point scale as follows: normal = 0, trivial = 1, mild = 2, moderate = 3, and severe = 4 for each stifle joint. Using this as a guide, we defined radiographic osteoarthritis grades of osteoarthritis score (OAS) from 0 to 10 (0 = no radiographic abnormalities identified; 1–3 = mild osteoarthritis; 4–6 = moderate osteoarthritis; 7–9 = severe osteoarthritis; 10 = most severe osteoarthritis). Changes in the osteoarthritis score (OAS) were evaluated by comparing preoperative OAS values with values obtained at the 8-week and 12-week postoperative rechecks.

## 3. Results

The results are summarized in Table 1. Five cats were included (three females and two males) in this study, with a mean body weight of 4.9 ± 1.5 kg (range: 3.1–6.6 kg) and a mean age of 3.4 ± 2.4 years (range: 1–6 years). The breeds included three European Shorthairs, one Exotic Shorthair, and one British Blue. Etiology was traumatic in three cases (high-rise syndrome in two cases and jumping off a closet in one case; *n* = 3, 60%). However, in the other two cases, there was no history of known trauma (*n* = 2, 40%). The mean time from the onset of clinical signs to surgery was 4.4 ± 3.1 weeks (range: 2–10 weeks). Three cats had previously received medical treatment with meloxicam and rest, and one with additional tramadol without favorable results. One cat had not received any previous medical treatment before surgery.

The mean preoperative TPA was 29.6° ± 4.85° (range: 24–37°). Two cases showed signs of OA at the time of initial evaluation. During arthrotomy, two cases of medial meniscal injury were observed, consisting of caudal horn folding; and in both cases, hemimeniscectomy was performed. All patients presented a complete rupture of the CrCL. Caudal cruciate ligament (CdCL) injury was not observed in any case. All osteotomies were stabilized with a six-hole CTWO 2.0 mm or 2.4 mm locking plate (2.4 mm: 3 cases; 2.0 mm: 2 cases), using three screws proximally and three screws distally to the osteotomy lines, along with a 0.8 mm closed-loop cerclage wire. The mean postoperative TPA was 4.9° ± 0.98° (range: 3.7–6.5°). The mean time to achieve complete bone healing (grade 4) was 9.65 ± 1.96 weeks (range: 8–12 weeks); at that time, all patients showed grade 0 (*n* = 2) or grade 1 intermittent lameness (*n* = 3). The radiographic follow-up period was twelve weeks for every patient. OAS values remained the same as those observed preoperatively in all cases except one, which showed a mild increase in OAS observed radiographically during follow-up (case 5: preoperative OAS:2, 12 weeks postoperative OAS:3). All patients were clinically assessed through orthopedic examination 6 months after surgery; at this point, the lameness score was zero, showing complete functional recovery of the limb.

No soft tissue or implant-related complications were recorded during the follow-up period. None of the patients showed signs of infection, implant loosening or breakage, fracture, or required implant removal. Stitches were removed two weeks after surgery, and all cases showed favorable progression without local or systemic alterations throughout clinical and radiographic follow-up. 

## 4. Discussion

In this short case series, excellent results were obtained following treatment of CrCL rupture using CTWO. The authors believe that a more in-depth discussion should be provided regarding the potential advantages that this technique may offer compared with treatments described in previous publications.

Conservative treatment using anti-inflammatory drugs and exercise restriction is a routine first approach in the management of CrCL rupture in cats. In 1998, Suter et al. [22] conducted an experimental study using cats as animal models of AO after cranial cruciate ligament transection (CCLT). The objectives of this study were to assess, over a one-year period, radiological changes, alterations in hind limb kinematics, ground reaction forces, and stifle stability. At the end of the study, preoperative gait parameters were recovered due to the development of new movement patterns of the stifle joint. However, this cannot be exclusively attributed to this adaptation process. Morphological changes within the joint have been described following cranial cruciate ligament transection. These include increased thickness of the joint capsule and medial collateral ligament [23,24]. Osteophyte formation and thinning of the articular cartilage have also been reported [22]. It is reasonable to suggest that these anatomical changes may also contribute to the adaptation of the stifle joint after cranial cruciate ligament rupture. Therefore, the development of osteoarthritis after ligament rupture may also play a role in joint stabilization. Scavelli et al. published the results of conservative treatment in 16 cats. Most showed good clinical progress after several months of restricted activity to allow for lesion healing. However, 80% continued to show instability in the femoropatellar joint and radiographic progression of OA [9].

Extracapsular techniques are usually used to treat cranial cruciate ligament rupture in cats [2,3,10,11]. In 2019, Boge et al. published epidemiological results in a group of 50 cats with cruciate ligament rupture [6], comparing outcomes of conservative versus surgical treatment with fabellopatellar suture using the Feline Musculoskeletal Pain Index (FMPI). Postoperative complications were recorded in 27.3% of surgically treated cats. Three of the six cats presented major complications and required a second surgery. They concluded that the conservatively treated group performed better regarding chronic pain. However, in the authors’ opinion, a comparison between the chronic pain scores of surgically treated animals without complications and of those treated conservatively would have provided valuable data. Additionally, they reported a 47% association between CrCL rupture and meniscal injury. It is consistent with other publications [5,7,8,13] and with our results (40% of cases). In dogs, the association between meniscal injury and early OA progression in the stifle is well established [25,26]. The authors believe this strong association is a compelling reason to favor surgical treatment. Boge et al. also reported that none of the surgically treated cats in their study had partial CrCL ruptures [6], which is also consistent with our results (all our patients presented complete CrCL ruptures).

The biomechanical behavior of different intra- and extracapsular suture configurations has been tested ex vivo [10,11,27,28]. It has been suggested that techniques aimed at achieving dynamic stabilization through the modification of femoropatellar joint biomechanics may offer superior functional outcomes compared with those providing passive stability through ligament replacement or augmentation in canine patients [14,20,29,30]. This concept has driven the development and use of dynamic stabilization techniques such as tibial tuberosity advancement (TTA) and tibial plateau leveling osteotomy (TPLO), which have been described in previous publications, as well as cTWO, the technique evaluated in the present study.

In ex vivo biomechanical studies, a lack of stabilization of the femorotibial joint with a transected CrCL was observed after performing TTA in this feline stifle model [31]. Treatment using TTA techniques has been reported for managing CrCL disease in cats as a standalone procedure [14,32], or concomitantly with medial patellar luxation (MPL) [15], with satisfactory outcomes. These results are limited by the small number of cases included in each study (one, two, and four, respectively) and they were associated with a significant complication rate. The simple translation of the TTA technique from dog to cat appears risky [31].

In 2018, Retournard et al. reported that TPLO failed to achieve stifle stabilization in terms of cranial tibial subluxation and the tibial rotation angle in CrCL-transected ex vivo feline models [31]. Nevertheless, they noted that the study had several limitations. In addition, Retournard et al.’s findings contradict the results of the study published in 2016 by Mindner et al., which reported good clinical results in cats treated with TPLO in a series of 11 cases [7]. However, they reported minor intraoperative complications in five cats and minor postoperative complications in three cats. Tamburro et al. also published successful results using TPLO in nine cats, reporting only one minor postoperative complication [13]. In both studies, OASs were established according to Freire et al. [19] using the same scoring scale we used in our study (absent = 0, slight = 1–3, mild = 4–6, moderate = 7–9, severe = 10). Mindner et al. reported an increase in OA evident in 3 out of 11 cats over a twelve-week period [7]. Tamburro et al. observed OAS progression in three out of nine cats [13]. In our study, we only observed a mild increase in OAS in one case during the follow-up (case 5: preoperative OAS:2, 12 weeks postoperative OAS:3).

Oxley et al. reported a modification of the CTWO technique using TPLO locking plates in dogs weighing 20–60 kg [21]. They compared the CTWO technique with TPLO and reported that both techniques are associated with similar complication rates and clinical outcomes when performed by experienced surgeons. In our study, we used the same modification of the CTWO technique reported by Oxley et al. and CTWO locking plates designed for use in dogs.

Combined TPLO and CTWO treatment was described in one case presenting a deformity of the proximal tibia with an exaggerated tibial plateau angle of approximately 75 degrees, with satisfactory results [16]. TPLO combination with CTWO has also been reported in dogs with excessive TPA [33]. Performing TPLO as a single procedure in these kinds of patients appears to be risky due to the excessive proximal fragment rotation, reduced bone contact at the osteotomy site, and subsequent predisposition to fixation failure. Other modifications of the CTWO technique have been reported in dogs to treat patients with excessive TPA, with successful results [34,35]. In the authors’ opinion, it would be of great interest to also investigate their application in cats with excessive TPA.

Cats with cranial cruciate ligament rupture present a significantly higher mean TPA (24.7 ± 4.5°) than cats without evidence of ligament injury (21.6 ± 3.7°) [36]. In our case series, the mean TPA was 29.6° ± 4.85° (range: 24–37°). This value, to our knowledge, exceeds those previously reported in the literature. Although whether an elevated TPA constitutes a risk factor for CrCL rupture in cats has not been determined, this finding reinforces the suitability of osteotomy techniques for joint stabilization. Studies with a larger number of cases are required to confirm this relationship.

Our findings were comparable to those reported by Aleksiewicz et al. [17], with satisfactory improvement of clinical signs, adequate bone healing, and absence of major complications following CTWO in feline patients. Although their study emphasized the use of a triangular saw guide to facilitate the osteotomy procedure and obtain more precise and orthogonal osteotomy cuts, the surgical technique described in the present study was performed without the assistance of a cutting guide. Nevertheless, the desired postoperative tibial plateau angle corrections established during preoperative planning were successfully achieved in all cases included in our study.

One of the limitations of the present study is that the assessment of osteoarthritis progression and lameness grading was based on subjective evaluation systems. Although previously described and widely used grading scales [18,19] were employed in an attempt to reduce observer bias, the interpretation of radiographic osteoarthritic changes and clinical lameness assessment inevitably retains a subjective component. Nevertheless, these evaluation methods have been extensively applied in previous veterinary orthopedic studies and currently represent commonly accepted tools for clinical assessment in feline orthopedic patients. 

## 5. Conclusions

Cranial tibial wedge osteotomy was performed in our feline patients without relevant technical difficulties. Successful and complete bone healing was observed in every case. No intraoperative or postoperative complications related to implants or soft tissues were recorded. All cats achieved complete functional recovery without lameness at the last recheck six months after surgery. These preliminary results suggest that CTWO may represent a feasible surgical alternative for the treatment of CrCL rupture in cats. However, considering the small sample size, retrospective nature of the study, lack of objective gait analysis, subjective osteoarthritis assessment, and relatively short follow-up period, the results of the present study should be interpreted with caution. Further prospective studies including a larger number of cases, objective functional evaluation, control groups, and longer follow-up periods are required to better assess the clinical application, long-term outcomes, and potential influence of this technique on osteoarthritis progression in feline patients.

## Figures and Tables

**Table 1:** Summary table of study results. Abbreviations: ESh: European Shorthair; m, male; f, female; nf, neutered female; w, weeks; kg, kilograms; y, years.

	Case Number	1	2	3	4	5
	Breed	ESh	Exotic	ESh	Blue British	ESh
	Sex	nf	m	f	nf	m
	Weight (kg)	5.8	3.7	3.1	6.6	5.6
	Age (y)	2	2	1	6	6
	Etiology	no previous trauma observed	traumatic (high-rise syndrome)	traumatic (high-rise syndrome)	no previous trauma observed	traumatic (jumping off a closet)
	Time since onset (w)	3	5	10	2	2
	Previous treatment	meloxicam rest	tramadol, meloxicam rest	meloxicam	meloxicam, rest	none
Physical, orthopedic and radiology exam	Lameness grade	3	4	3	4	4
	Limb affected	right	right	right	right	left
	Drawer test	positive	positive	positive	positive	positive
	Joint inflammation	marked	marked	moderate	moderate	moderate
	Knee rom	normal	mildly reduced	markedly reduced	normal	normal
	Crepitus	no	yes	yes	no	yes
	OAS	0	0	6	0	2
	Joint effusion	yes	yes	yes	yes	yes
	Preoperative TPA	24	37	24	31	32
Surgery	CrCL	complete rupture	complete rupture	complete rupture	complete rupture	complete rupture
	CdCL	intact	intact	intact	intact	intact
	Medial meniscus	intact	intact	folded	intact	folded
	Lateral meniscus	intact	intact	intact	intact	intact
	CTWO plate size	2.4 mm CTWO plate	2.0 CTWO locking plate	2.0 CTWO locking plate	2.0 CTWO locking plate	2.4 CTWO locking plate
	Postoperative TPA	4.2	6.5	5.4	3.7	4.7
Follow-up	Weeks after surgery	8	8	8	8	8
	Lameness grade	0	0	1	2	1
	Bone healing grade	4	4	4	2	2
	OAS	0	0	6	0	2
	Weeks after surgery	12	12	12	12	12
	Lameness grade	0	0	1	1	1
	Bone healing grade	4	4	4	4	4
	OAS	0	0	6	0	3
	Lameness grade 6-month postoperative clinical recheck	0	0	0	0	0

## Data Availability

The original contributions presented in this study are included in the article. Further inquiries can be directed to the corresponding author.
